# Disruption of Annexin II /p11 Interaction Suppresses Leukemia Cell Binding, Homing and Engraftment, and Sensitizes the Leukemia Cells to Chemotherapy

**DOI:** 10.1371/journal.pone.0140564

**Published:** 2015-10-14

**Authors:** Anilkumar Gopalakrishnapillai, E. Anders Kolb, Priyanka Dhanan, Robert W. Mason, Andrew Napper, Sonali P. Barwe

**Affiliations:** Nemours Center for Childhood Cancer Research, Alfred I. duPont Hospital for Children, Wilmington, DE, 19803, United States of America; University of Sydney, AUSTRALIA

## Abstract

The bone marrow microenvironment plays an important role in acute lymphoblastic leukemia (ALL) cell proliferation, maintenance, and resistance to chemotherapy. Annexin II (ANX2) is abundantly expressed on bone marrow cells and complexes with p11 to form ANX2/p11-hetero-tetramer (ANX2T). We present evidence that p11 is upregulated in refractory ALL cell lines and patient samples. A small molecule inhibitor that disrupts ANX2/p11 interaction (ANX2T inhibitor), an anti-ANX2 antibody, and knockdown of p11, abrogated ALL cell adhesion to osteoblasts, indicating that ANX2/p11 interaction facilitates binding and retention of ALL cells in the bone marrow. Furthermore, ANX2T inhibitor increased the sensitivity of primary ALL cells co-cultured with osteoblasts to dexamethasone and vincristine induced cell death. Finally, in an orthotopic leukemia xenograft mouse model, the number of ALL cells homing to the bone marrow was reduced by 40–50% in mice injected with anti-ANX2 antibody, anti-p11 antibody or ANX2T inhibitor compared to respective controls. In a long-term engraftment assay, the percentage of ALL cells in mouse blood, bone marrow and spleen was reduced in mice treated with agents that disrupt ANX2/p11 interaction. These data show that disruption of ANX2/p11 interaction results in reduced ALL cell adhesion to osteoblasts, increased ALL cell sensitization to chemotherapy, and suppression of ALL cell homing and engraftment.

## Introduction

Acute lymphoblastic leukemia (ALL) is a systemic disease characterized by proliferation and accumulation of leukemic cells within the bone marrow. Leukemic cells egress from the bone marrow, enter the blood circulation and populate organs such as liver, spleen, lymph nodes, thymus and the central nervous system. It is believed that while circulating leukemic cells are sensitive to current therapies, survival of a sub-population of leukemic cells may be favored by localization to the hematopoietic stem cell (HSC) niche within the bone marrow, ultimately leading to relapse. Bone marrow is the principal site of relapse, underlining the significance of the bone marrow microenvironment in the progression and maintenance of malignancy [1]. Adhesion of leukemic cells to cells within the HSC niche increases their survival and maintains them in a quiescent state (similar to HSCs), thereby inducing resistance to chemotherapeutic drugs, which mostly target proliferating cells [2, 3].

The adhesive interactions between HSCs and osteoblasts in the bone marrow niche are mediated by annexin II (ANX2), which acts as a ligand for adhesion, homing and engraftment of HSCs following transplantation [4]. ANX2 is a calcium-dependent phospholipid-binding protein that forms a heterotetramer composed of two subunits of ANX2 linked together by a dimer of p11. The p11 protein (also called S100A10) is a member of the S100 EF-hand superfamily of calcium-binding proteins and serves as a regulatory subunit of ANX2T. ANX2/p11 heterotetramer (ANX2T) plays a role in malignancy by allowing metastatic prostate cancer cells [5] and multiple myeloma cells [6] to reach the bone marrow.

p11 alone can associate with the plasma membrane in the absence of ANX2 and facilitate plasmin activation and invasiveness in colorectal cancer cells [7]. Upregulation of p11 has been reported in several cancers including renal cell carcinoma [8], squamous non-cell lung cancer [9], anaplastic large cell lymphoma [10], and pediatric intracranial ependymoma [11]. However, the role of the p11 in the development and progression of acute lymphoblastic leukemia has not been intensely investigated.

A function-blocking anti-ANX2 antibody or a peptide corresponding to the N-terminal amino acids 1–12 of ANX2 containing the minimal p11 binding site prevents the homing and engraftment of HSCs [4], prostate cancer cells [5], and multiple myeloma cells [6] to the bone marrow in irradiated mice. A number of small molecule inhibitors (1-substituted 4-aroyl-3-hydroxy-5-phenyl-1 H-pyrrol-2(5H)-one analogs) of the ANX2/p11 interaction (ANX2T inhibitor) that are able to disrupt the physiological complex in cell lysates have been identified by structure-based virtual screening [12]. In this manuscript, we show that p11 is upregulated in bone marrow from relapsed B-cell ALL (B-ALL) patients, and p11 on the ALL cell surface mediates adhesion of B-ALL cells to osteoblasts. Treatment with anti-ANX2 antibody or ANX2T inhibitor resulted in suppression of homing and engraftment of ALL cells to the bone marrow in a leukemia xenograft mouse model. Moreover, primary B-ALL cells co-cultured with osteoblasts were sensitized to standard chemotherapeutics in the presence of ANX2T inhibitor.

## Methods

### Cell lines, patient samples and amplification of patient cells by passaging in mice

RS4;11 (CRL-1873), REH (CRL-8286) and Saos-2 (HTB-85) cells were obtained from American Type Culture Collection (ATCC), Manassas, VA. Nalm6 cells were purchased from DSMZ-German Collection of Microorganisms and Cell Cultures, Braunschweig, Germany. AG09390 and AG15007 cells were from Coriell Institute for Medical Research, Camden, NJ. Leukemia or lymphoblastoid cell lines were cultured in RPMI culture medium supplemented with 10% fetal bovine serum (FBS), 2 mM/L L-glutamine, 25 U/mL penicillin, and 25 μg/mL streptomycin. Saos-2 cells were cultured in DMEM/F12 (1:1) with supplements described above.

Mononuclear cells were isolated from peripheral blood of healthy adult volunteers. Primary B-ALL cells isolated from bone marrow aspirates or peripheral blood of patients treated at Nemours/Alfred I. duPont Hospital for Children, are banked by the Nemours BioBank. Samples were collected under a Nemours Delaware Institutional Review Board (IRB) protocol approved by the Nemours Office of Human Subjects Protection.

Since the number of cells obtained from patients was limited, primary B-ALL cells were amplified by passaging in mice as described previously [13], with some modifications. Non-obese diabetic/severe combined immunodeficient (NOD/SCID) mice that also harbor null alleles for interleukin-2-receptor gamma gene (IL2-Rγ) and the major histocompatibility complex (MHC) class I molecule beta2-microglobulin gene (B2m) (referred to as NSG-B2m mice, Jackson Laboratories) were used. Primary B-ALL cells (1–10 x 10^6^) were transplanted into NSG-B2m mice via tail-vein injections. Mice were maintained in the Nemours Life Science Center following the guidelines established by the Nemours Institutional Animal Care and Use Committee. Disease progression was monitored by flow cytometry of mouse peripheral blood drawn weekly. Mice were sacrificed by carbon-di-oxide asphyxiation using a method consistent with AVMA recommendations when they exhibited disease symptoms: increased leukemic burden, persistent weight loss or hind-limb paralysis. Following sacrifice, leukemic cells were harvested from the spleens and enriched by Ficoll gradient centrifugation. Flow cytometry (described below) confirmed that more than 95% of the cell population consisted of human leukemic cells. These mouse-passaged primary B-ALL cells were utilized for chemoresistance, homing and engraftment assays described below. All studies involving mice were approved by the Nemours Institutional Animal Care and Use Committee.

### Antibodies and reagents

Mouse monoclonal antibodies against ANX2, p11 and β-catenin were obtained from BD Biosciences (San Jose, CA). Rabbit polyclonal GAPDH antibody and horseradish peroxidase conjugated secondary antibodies were from Cell Signaling Technology (Lexington, KY). FITC-conjugated anti-mouse secondary antibody was purchased from Jackson ImmunoResearch Laboratories (West Grove, PA). Alexa Flour 488 conjugated secondary antibody was from Life Technologies (Grand Island, NY). FITC-conjugated human CD45 and APC-conjugated mouse CD45 antibodies were obtained from eBioscience (San Diego, CA). Osteocalcin antibody was from Santa Cruz Biotechnology (Dallas, TX) and CD45 antibody was from Leica (Buffalo Grove, IL). Isotype-matched control IgG, dexamethasone and vincristine sulfate were obtained from Sigma-Aldrich (St. Louis, MO). 5-Benzyl-4-methyl-2-(toluene-4-sulfonylamino)-thiophene-3-carboxylic acid amide was purchased from Asinex (Winston-Salem, NC).

### Flow cytometry

Peripheral blood was stained with FITC-conjugated anti-human CD45 and APC-conjugated anti-mouse CD45. Following lysis of red blood cells, the number of cells stained specifically for either antibody within a cell population gated for lymphocytes (based on their forward and side scatter parameters) was determined by flow cytometry. The percentage of human cells in mouse peripheral blood was calculated using this formula–(No. of human CD45+ cells)/(No. of human CD45+ cells + No. of mouse CD45+ cells) x 100.

ALL cells were fixed in 4% paraformaldehyde for 10 min at 37°C. Cells were blocked in 0.5% bovine serum albumin in phosphate-buffered saline (PBS) and incubated with primary antibody for 1 h at room temperature. After washing, cells were incubated with FITC-conjugated secondary antibody for 30 min at room temperature. After washing, cells were resuspended in PBS and analyzed on a flow cytometer, BD Accuri C6, BD Biosciences. A sample stained with secondary antibody alone was used as a control for non-specific staining.

### Quantitative real-time PCR

Total RNA was extracted using Trizol reagent from Invitrogen (Carlsbad, CA). First-strand cDNA was generated from total RNA using the iScript cDNA synthesis kit (Bio-Rad, Hercules, CA) according to the manufacturer’s protocol. Quantitative PCR reaction was performed using SYBR qPCR master mix on ABI7900HT (Applied Biosystems, Grand Island, NY). Primer sequences for ANX2: Forward primer 5’-GAAACAGCCATCAAGACCAAAGG-3’, Reverse primer 5’-TGGTAGGCGAAGGCAATATCC-3’, p11 Forward primer 5’- AGGAGTTCCCTGGATTTTTGG-3’, Reverse primer 5’- TACACTGGTCCAGGTCCTTCATTA-3’, glucose-6-phosphate dehydrogenase (GAPDH) Forward primer 5’-GCTGTCCAACCACATCTCCTC-3’, Reverse primer 5’-TGGGGCCGAAGATCCTGTT-3’. Relative gene expression was calculated by Applied Biosystems SDSv2.4 software using the comparative threshold method and the values were normalized against the endogenous control GAPDH.

### Immunoblotting and immunoprecipitation

Cells were lysed in a buffer containing 20 mM Tris-HCl (pH 7.4), 100 mM NaCl, 1% v/v Triton X-100, 1 mM EDTA, 1 mM EGTA, 1 mM sodium glycerol phosphate, 1 mM sodium orthovanadate, 1 mM PMSF, and 5 μg/ml each of antipain, pepstatin and leupeptin. The lysates were sonicated and clarified by centrifugation at 16,000 g for 10 min at 4°C. Total protein in cell lysates was estimated by Bio-Rad DC Reagent following manufacturer’s instructions. One hundred μg of total protein was resolved by SDS-PAGE, transferred to nitrocellulose membrane, and immunoblotted using a primary antibody, followed by HRP-conjugated secondary antibody diluted in Tris-buffered saline containing 5% w/v non-fat dried milk and 0.1% v/v Tween-20. Enhanced chemiluminescent lighting system, PerkinElmer Life Sciences (Boston, MA) was used for detection.

For immunoprecipitation, Nalm6 cells were treated with DMSO or ANX2T inhibitor for 6 h prior to lysis as described above. Lysates corresponding to 1 mg of total protein were incubated overnight with anti-p11 antibody (25 μg per sample) coupled to Protein A Mag Agarose beads, GE^®^ Biosciences (Pittsburg, PA), via rabbit anti-mouse antibody. The beads were washed and the proteins bound to the beads were resolved on SDS-PAGE and detected by immunoblotting as described above.

### shRNA knockdown and transfections

For p11 knockdown, the p*Silencer* 5.1-U6 Retro vector from Ambion, Life Technologies, containing specific siRNA targeting sequences (Seq 1—AGGAGGACCTGAGAGTACT or Seq 2—GCAGAAGGGAAAGAAGTAG) was transfected into Nalm6 cells by nucleofection with Amaxa Nucleofector, Lonza (Basel, Switzerland) using solution T and program C-005 following manufacturer’s protocol. Forty-eight hours after transfection cells were used for immunofluorescence or in a cell adhesion assay.

### Immunofluorescence

Cells were layered gently on glass slides coated with poly-L-Lysine (Sigma-Aldrich). Once adhered, the cells were fixed using 2% paraformaldehyde for 30 min at room temperature. Fixed, non-permeabilized cells were incubated with primary antibody, followed by Alexa Flour 488 conjugated secondary antibody and mounted in ProLong gold antifade reagent (Life Technologies). Single confocal sections were obtained using a Leica TCS SP5 Confocal Microscope (Leica Microsystems) with the same laser power, gain and offset settings. The average fluorescence intensity from at least 100 cells was quantitated using LAS AF software (Leica Microsystems).

### Cell adhesion assay

Cell adhesion was assayed as described [4]. Briefly, ALL cells were labeled with 2.5 μg/ml of lipophilic dye carboxyfluorescein diacetate (Molecular Probes—Life Technologies) for 30 min at 37°C. The excess dye was removed by washing with PBS and the cells were rested for 30 min in dark to quench non-specific background. Labeled cells (1 x 10^5^) were loaded on to osteoblasts plated in 96-well culture plates in the presence of anti-ANX2 antibody or isotype-specific control antibody. Similar assay was performed in the presence of ANX2T inhibitor or vehicle. After centrifugation at 100 g for 5 min and incubation for 30 min at 4°C, non-adherent cells were removed by washing with PBS. A set of wells was left untouched and considered as ‘input’. The fluorescence intensity of input was similar in the presence or absence of antibody or ANX2T inhibitor. The fluorescence corresponding to the number of adherent ALL cells was quantified by using a Victor X4 plate reader (PerkinElmer). Binding efficiency was calculated as the percentage fluorescence of adherent cells with respect to the total fluorescence of the “input”.

### Chemoresistance co-culture assay

Mouse passaged primary ALL cells with or without co-culture with Saos-2 cells were maintained in RPMI supplemented with 20% FBS and penicillin streptomycin as described under “Cell lines and patient samples”. Cells were treated with 1 μM dexamethasone or 10 nM vincristine in the presence or absence of ANX2T inhibitor (100 μM) for 24 h. ALL cells were removed from adherent osteoblasts by vigorous pipetting and the number of viable cells in an ungated population was determined by propidium iodide exclusion using a BD Accuri C6 flow cytometer. The number of viable cells was expressed as a percentage of control cells co-cultured with Saos-2 osteoblasts in the presence of DMSO (used as compound delivery vehicle). For determination of osteoblast cell viability, CellTiter-Blue reagent from Promega (Madison, WI) was used following manufacturer’s protocol.

### Immunohistochemistry

Femurs isolated from NSG-B2m mice transplanted one week prior with 10 million ALL cells were fixed in 10% NBF for 24–48 hours. The femurs were demineralized in RegularCal™ Immuno (BBC Biochemical, Mt. Vernon, WA) and checked for end point decalcification before washing in running water. The samples were processed on a routine clock and paraffin embedded in Histoplast LP (Thermo Fisher Scientific, Fremont, CA). All samples were cut at 5 μm on a RM2255 microtome (Leica Biosystems) and floated onto Superfrost^®^ Plus Gold slides (Thermo Fisher Scientific). The sections were heat immobilized for 60 minutes at 60°C, and stored at -20°C until ready to stain. Slides were equilibrated to room temperature then deparaffinized in xylene and re-hydrated to deionized water (ddH_2_0) prior to antigen retrieval. Heat retrieval was performed in a 60°C oven with a sodium citrate buffer at pH 6 overnight, and allowed to cool to room temperature. The slides were placed on Bond RX IHC stainer (Leica Biosystems) for double staining. The first antibody, osteocalcin was applied to the slides; then the slides were systematically moved through the Bond Polymer Refine Detection solutions consisting of a post primary solution, a peroxide block, a polymer and DAB chromogen. After which the second antibody, CD45, was applied and systematically moved through the Bond Polymer Refine Red Detection solutions consisting of a post primary AP, a polymer AP, Red Refine chromogen and finally counter stained with hematoxylin. When finished, the slides were dehydrated, cleared and mounted in Permount^®^ (Thermo Fisher Scientific). Images were captured using Nikon Eclipse 80i microscope (Nikon Instruments, Melville, NY).

### Short-term homing assay

Anti-ANX2 antibody, anti-p11 antibody or control IgG (each at 10 μg/Kg) was injected into the tail vein of NSG-B2m mice, followed after 1 h by an injection of 10 x 10^6^ ALL cells. In a separate experiment, ANX2T inhibitor (10 mg/Kg) or vehicle (5% dextrose) was used instead of the antibodies. Peripheral blood was drawn before mice were sacrificed twenty-four hours post transplantation. Femurs were flushed to collect bone marrow cells. Spleen and liver tissue was harvested and homogenized to generate a cell suspension. The percentage of human cells in mouse tissue was determined by flow cytometry using FITC-conjugated anti-human CD45 and APC-conjugated anti-mouse CD45 as described above.

### Engraftment assay

Antibody, ANX2T and ALL cell injections were performed as described above for the short-term homing assay, except the number of ALL cells injected was 1x10^6^. In addition, a second dose of anti-ANX2 antibody or two more doses of ANX2T inhibitor were administered on the following days via tail-vein injection. Engraftment was determined using flow cytometry to estimate the proportion of human cells in mouse peripheral blood two weeks post cell injection.

### Statistical Analysis

Paired T-Test was used to evaluate the differences between average of two groups using data from at least three independent experiments and a minimum P value of < 0.05 was considered statistically significant.

## Results

### p11 is upregulated in pediatric B-cell acute lymphoblastic leukemia

**Fig 1:**
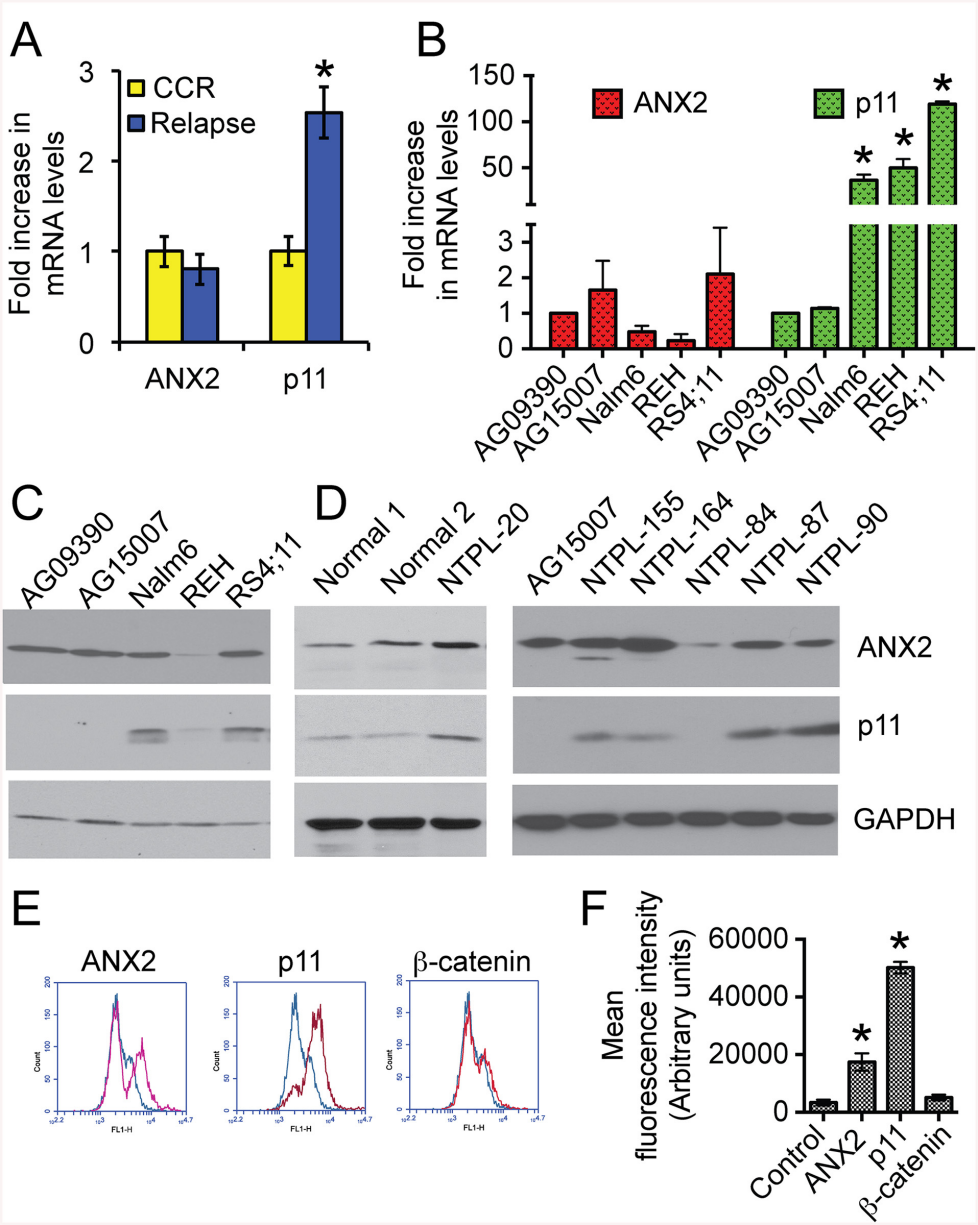
p11 is upregulated at mRNA and protein levels and is localized to the cell surface in ALL cells. **A)** Analysis of microarray data from GSE7440 showing fold-increase in transcript levels of ANX2 and p11. CCR = patients under continuous complete remission for more than four years (n = 28). Relapse = patients who relapsed within three years of initial diagnosis (n = 31). Error bars denote Standard Deviation (SD) of the Mean. Asterisk indicates statistical significance with a confidence limit of 95%. **B)** Quantitative RT-PCR data showing mRNA levels of ANX2 and p11 in pediatric normal immortalized B-cells (AG09390 and AG15007) and pediatric (Nalm6, REH) or adult (RS4;11) pre B-ALL cell lines. Error bars denote SD of the Mean from four independent experiments. Asterisks indicate statistical significance with a confidence limit of 95%. **C)** Immunoblots showing ANX2 and p11 levels in the indicated cell lines. GAPDH was used as a loading control. Representative blots from three independent experiments are shown. **D)** Representative immunoblots showing ANX2 and p11 levels in lysates from mononuclear cells isolated from healthy volunteers (Normal 1 and 2), or from leukemic cells from pediatric patients. Samples were collected using IRB approved protocols. **E)** Flow cytometry profiles of fixed, non-permeabilized RS4;11 cells incubated with (red trace) or without (blue trace) addition of the indicated primary antibodies. **F)** Mean fluorescence intensity was plotted from three independent experiments. Error bars denote SD of the Mean. Asterisks indicate statistical significance (P < 0.01).

Microarray data is available from a genomics study (GSE7440) using National Cancer Institute-defined high-risk pediatric B-precursor ALL patients treated on the Children’s Oncology Group 1961 protocol [14]. In this study, bone marrow blast samples were obtained from 59 patients at diagnosis. Gene expression profiles from patients who remained in continuous complete remission for more than 4 years (CCR; n = 28) were compared with those from patients who relapsed within 3 years of initial diagnosis (Relapse; n = 31). Our analysis of the microarray data revealed that ANX2 mRNA levels were not significantly different (*P* = 0.4) between the two groups (Fig 1A). However, mRNA levels of p11 were elevated 2.5-fold in patients with early relapse, indicating that p11 expression is associated with and could be predictive of relapse (*P* = 0.0251). We next compared mRNA and protein levels of ANX2 and p11 between relapsed ALL cell lines and pediatric B lymphocytes immortalized by Epstein-Barr virus transformation (Coriell Institute for Medical Research), which are routinely used as control B-cells [15, 16]. Compared to the control cells (AG09390 and AG15007), an increase of 35–120 fold in the p11 mRNA levels was observed in all three human B-cell precursor leukemia cell lines established from ALL patients at first relapse (Fig 1B). Western blot analysis of cell lysates showed that p11 protein levels were also higher in the relapsed ALL cells (Nalm6 and RS4;11) than in control cells (Fig 1C). REH cells possess high levels of p11 mRNA but very low levels of the protein probably because of reduced ANX2 protein. It has been shown earlier that p11 mRNA is detected in ANX2 knockout mice, however p11 protein is very low, indicating that ANX2 is required for p11 protein expression [17]. ANX2 mRNA and protein levels were not increased in ALL cell lines (Fig 1B and 1C). p11 protein was also higher in five out of six primary B-ALL samples obtained from the Nemours Biobank compared to peripheral blood mononuclear cells isolated from the blood of healthy donors or compared to immortalized B-cell lines (Fig 1D). The levels of ANX2 protein did not show consistent changes between normal cells and patient samples. NTPL-84 had reduced levels of p11 likely because of reduced ANX2 protein similar to REH cells.

### p11 protein is localized to the cell surface and mediates binding of ALL cells to osteoblasts

Although p11 is a secreted soluble protein, it is localized to the cell surface either as a hetero-tetramer with ANX2 or by itself [7]. In our study, flow cytometry showed that p11 is localized to the exterior of the cell membrane of >90% of RS4;11 cells (Fig 1E; p11 red trace and Fig 1F). Some of these cells (~30%) also have ANX2 staining on the cell surface. As a control for non-permeabilized fixation, cells were stained with an antibody against the intracellular protein β-catenin. As expected, no specific cell surface staining for β-catenin was detected (Fig 1F). These data indicate that p11 is localized to the exterior of ALL cells.

**Fig 2:**
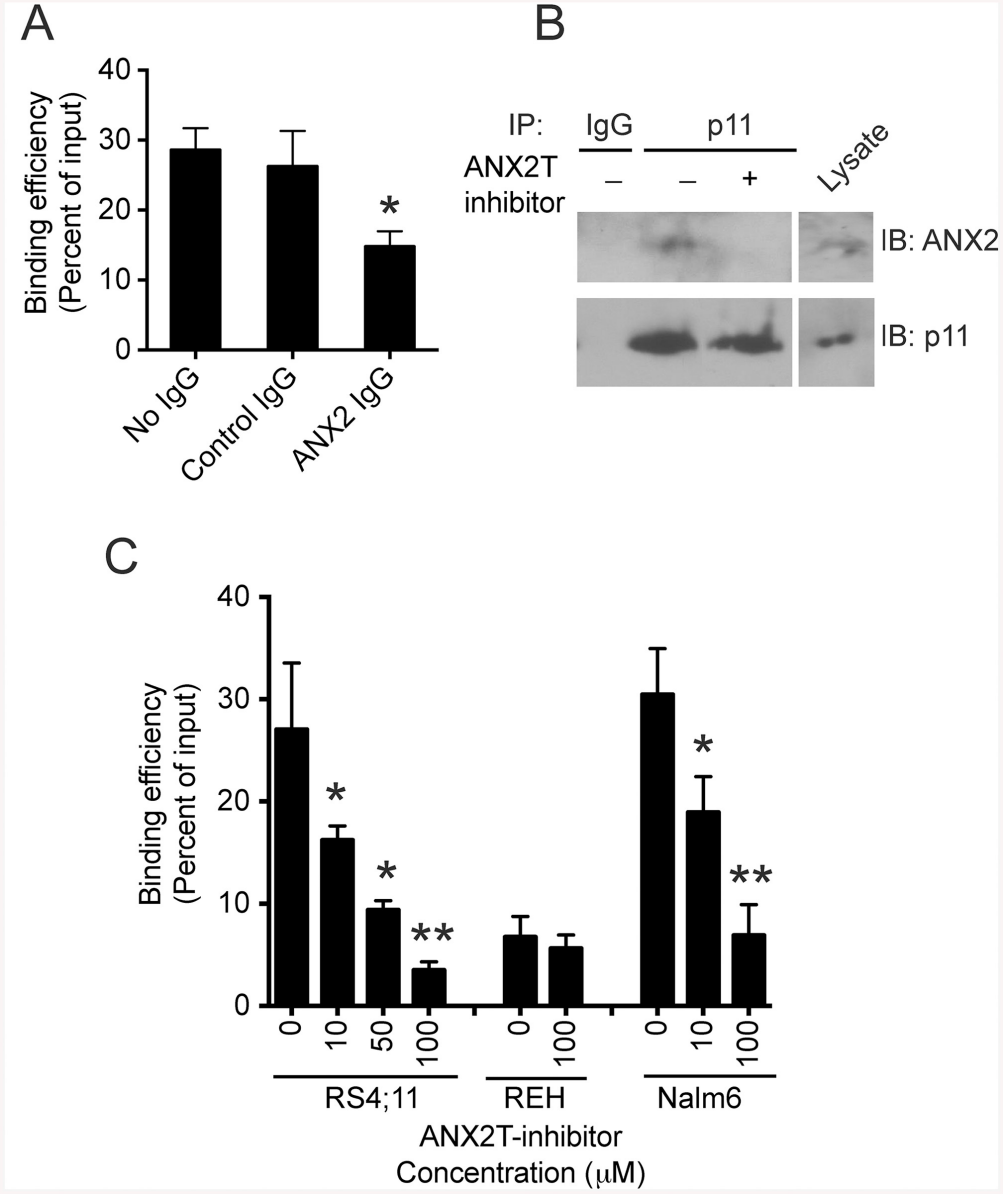
ANX2/p11 interaction mediates binding of ALL cells to osteoblasts. **A)** Cell adhesion assay showing the percentage of RS4;11 cells bound to Saos-2 monolayer with respect to the input, which was considered 100%. Error bars denote SD of the Mean from three independent experiments. Asterisk indicates statistical significance, P < 0.01. **B)** Immunoprecipitates using control IgG or anti-p11 antibody from lysates of Nalm6 cells treated with or without ANX2T inhibitor (50 μM) were blotted using ANX2 or p11 antibodies. Cell lysate corresponding to 10% of the total protein input in the co-immunoprecipitation assay was loaded in the lane denoted as “Lysate”. **C)** Graph shows the percentage of cells that bound to Saos-2 monolayer in the presence or absence of ANX2T inhibitor with respect to the input. Error bars indicate SD of the Mean from three to four independent experiments. Note the dose dependent decrease in the percentage of bound Nalm6 and RS4;11 cells post treatment with ANX2T inhibitor (*P < 0.05, **P < 0.01).

A recent study showed that p11 forms a molecular bridge with ANX2, which mediates binding of breast cancer cells to endothelial cells [18]. Similar to endothelial cells, osteoblasts within the bone marrow have large amounts of ANX2 on their cell surface [4]. Therefore, we decided to determine whether ANX2/p11 on cell surfaces plays a significant role in mediating adhesive interaction between ALL cells and osteoblasts. RS4;11 cells were cultured on confluent monolayers of Saos-2, a human osteosarcoma cell line commonly used as a model system for osteoblasts [4], in the presence or absence of anti-ANX2 antibody. The function-blocking Anti-ANX2 antibody has been shown previously to prevent adhesive interaction between osteoblasts and HSCs [4]. We observed a 50% reduction in the binding of RS4;11 cells to Saos-2 cells in the presence of anti-ANX2 antibody compared to control IgG or no IgG (Fig 2A). Similar results were obtained using Nalm6 cells (data not shown). To determine if ANX2/p11 interaction was involved in cell-cell binding, we used a small-molecule inhibitor to disrupt interaction between ANX2 and p11. The most potent ANX2T inhibitor identified by the Dekker lab is 5-Benzyl-4-methyl-2-(toluene-4-sulfonylamino)-thiophene-3-carboxylic acid amide (IC_50_ ~2.1 μM), which is commercially available from Asinex [12]. First, we tested whether ANX2T inhibitor can disrupt ANX2/p11 interaction in ALL cells. In a co-immunoprecipitation assay, the amount of ANX2 protein in immunoprecipitates using anti-p11 antibody was reduced by 90% in lysates from Nalm6 cells treated with ANX2T inhibitor (Fig 2B), indicating that the ANX2T inhibitor indeed prevents interaction between ANX2 and p11. Treatment with this ANX2T inhibitor reduced binding of Nalm6 and RS4;11 cells to osteoblasts in a dose-dependent manner (Fig 2C). The binding of REH cells, which do not have p11 protein, was unaffected by ANX2T inhibitor. Interestingly, a comparison between the binding efficiencies of the three cell lines revealed that the binding of REH cells was greatly reduced compared to RS4;11 and Nalm6 cells (Fig 2C). Taken together, these data indicate that p11 on ALL cell surface mediates binding between ALL cells and osteoblasts.

**Fig 3:**
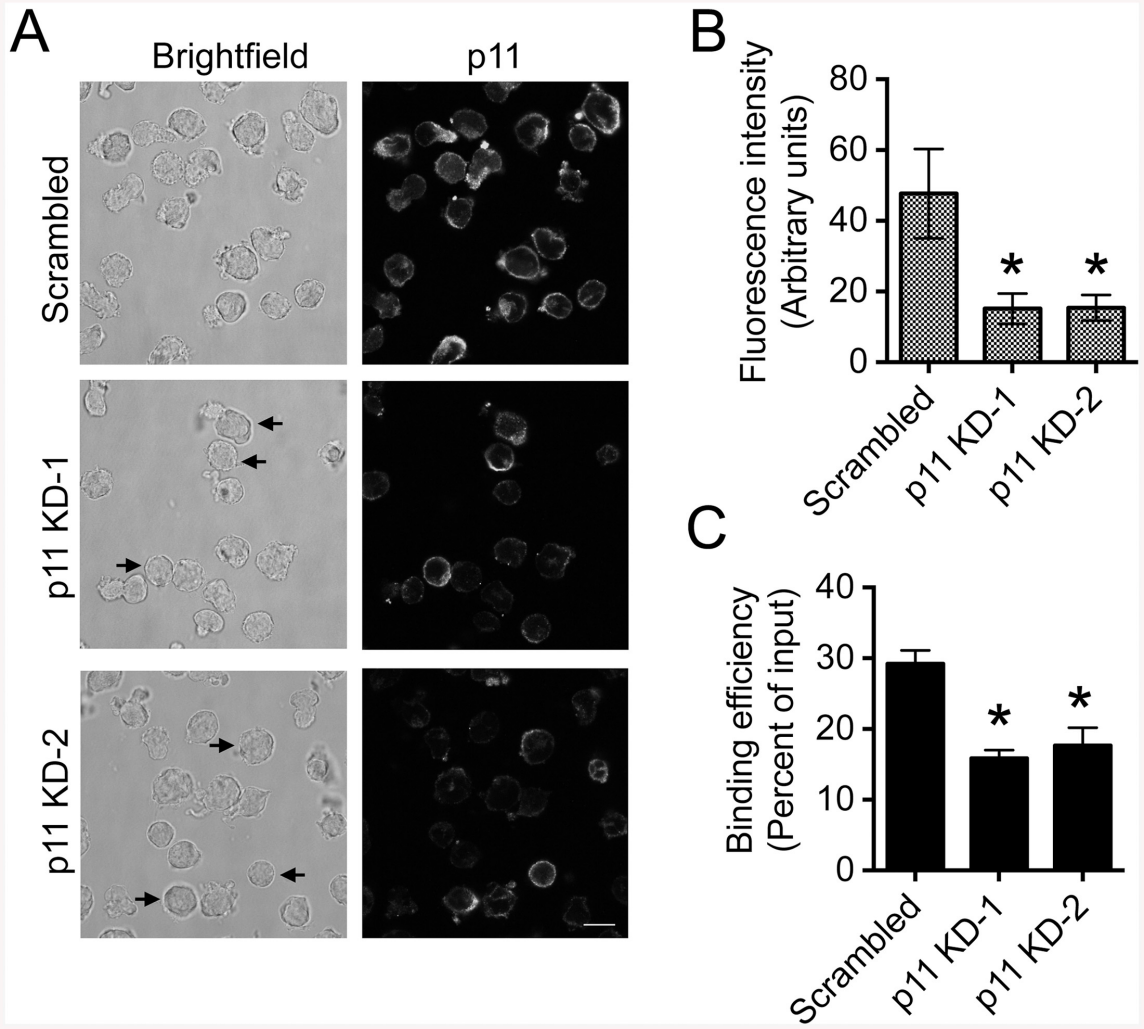
Knockdown of p11 reduced ALL cell binding to osteoblasts. **A)** Nalm6 cells were transfected with either scrambled or p11-specific shRNA (sequence 1 or sequence 2). Forty-eight hours post transfection, cells were plated on poly-lysine coated slides, fixed, stained with anti-p11 antibody followed by Alexa Flour 488-conjugated anti-mouse secondary antibody, and visualized by confocal microscopy. Scale bar = 10 μm. Representative images in gray scale are shown. **B)** The mean fluorescence intensity corresponding to p11 protein levels was determined from 100 cells per condition. The error bars denote SD of the Mean (*P < 0.01). **C)** Transfected cells were subjected to adhesion assay as described in Materials and Methods. Error bars denote SD of the Mean from two independent experiments in quadruplicates. Asterisks indicate statistical significance (*P < 0.001).

To confirm the role of p11 in mediating the binding between ALL cells and osteoblasts, Nalm6 cells were transiently transfected with a plasmid harboring shRNA against p11. Two different shRNA targeting sequences were used. Immunofluorescence of scrambled shRNA transfected, non-permeabilized Nalm6 cells showed p11 localization to the cell surface (Fig 3A) consistent with the flow cytometry data (Fig 1E). Cells transfected with p11 shRNA had diminished surface staining. The average transfection efficiency achieved was 75% and the p11 protein was reduced by 67–68% as determined by quantitation of immunofluorescence using anti-p11 antibody (Fig 3B). The levels of ANX2 protein did not change in the p11 knockdown cells (data not shown). In a cell adhesion assay, the binding of Nalm6 cells with p11 knockdown was reduced to 54–60% of scrambled (Fig 3C), confirming that p11 is involved in mediating binding between ALL cells and osteoblasts.

### ANX2T inhibitor increases the sensitivity of primary ALL cells co-cultured with osteoblasts to dexamethasone and vincristine

**Fig 4:**
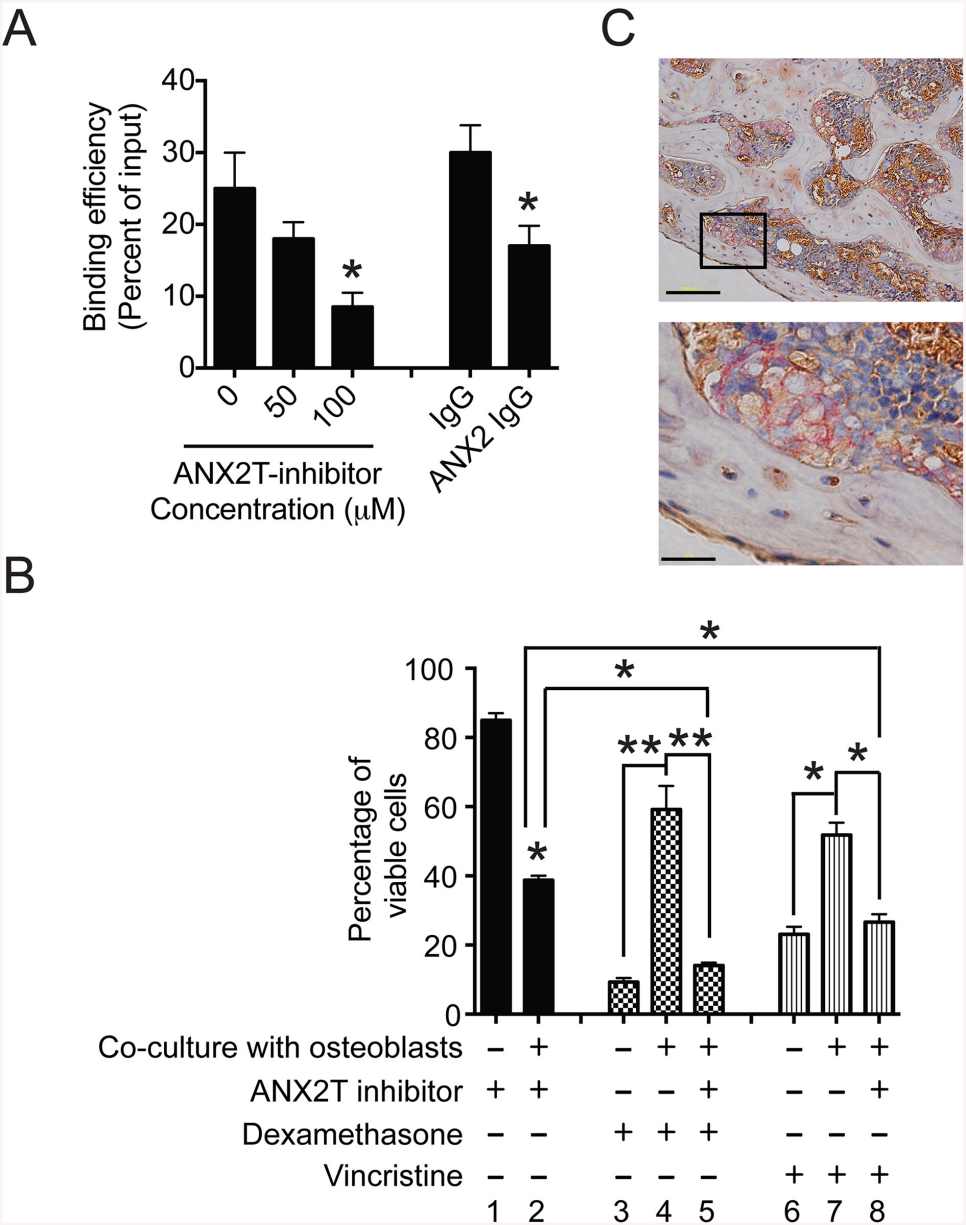
ANX2T inhibitor sensitizes primary ALL cells co-cultured with osteoblasts to dexamethasone and vincristine. **A)** Graph shows the percentage of NTPL-20 cells that bound to Saos-2 monolayer in the presence or absence of ANX2T inhibitor or anti-ANX2 antibody with respect to the input. Error bars indicate SD of the Mean from three independent experiments. Asterisks denote P < 0.01. **B)** Primary ALL cells (NTPL-20) were either plated on tissue culture plastic or Saos-2 monolayers and treated with 1 μM dexamethasone or 10 nM vincristine. Twenty-four hours post incubation, leukemic cells were collected by vigorous pipetting and stained with propidium iodide. The percentage of viable cells in the population was determined by flow cytometry based on exclusion of propidium iodide. Graph represents the percentage of viable cells in each sample normalized to the control (cells plated on osteoblasts in the presence of DMSO considered as 100%). Asterisks indicate statistical significance (*P < 0.05, **P < 0.01). **C**) Representative images of histological sections of femur from a mouse transplanted with NTPL-20 cells. Dual immunohistochemistry staining was performed showing osteocalcin (osteoblast specific marker, in brown) and CD45 (marker for ALL cells, in red). The black square in the upper panel marks the region of the slide, which is magnified in the lower panel. Scale bars = 100 μm. Similar data was obtained in three different mice.

Adhesion of ALL cells to stromal cells makes them more resistant to chemotherapeutics [2]. Therefore, we tested whether disruption of ALL cell interaction with osteoblasts by treatment with ANX2T inhibitor could sensitize ALL cells to commonly used chemotherapeutics such as dexamethasone and vincristine. For this purpose, we utilized primary ALL cells (NTPL-20), which express p11 (Fig 1D). NTPL-20 cells showed reduced binding to osteoblasts in the presence of ANX2T inhibitor or anti-ANX2 antibody compared to vehicle or isotype-specific control antibody respectively (Fig 4A).

ANX2T inhibitor showed minimal change in cell viability when cells were cultured on tissue culture plastic (Fig 4B, bar 1). Similarly, ANX2T inhibitor did not alter the viability of osteoblasts (88±5% compared to 100% of control) in a CellTiter-Blue cell viability assay. However, this inhibitor induced cytotoxicity in cells co-cultured with osteoblasts (Fig 4B, bar 2). Similarly, NTPL-20 cells co-cultured with osteoblasts were less sensitive to cell death induced by either dexamethasone (Fig 4B, compare bars 3 and 4) or vincristine (Fig 4B, compare bars 6 and 7), indicating that the osteoblasts have a protective effect on ALL cells, as described previously in the case of acute myeloid leukemia cells [19]. Co-cultured ALL cells treated with both dexamethasone and ANX2T inhibitor showed reduced viability compared to cells treated with either compound alone (Fig 4B, compare bar 5 with bars 2 and 4). Similar results were obtained in cells treated with vincristine in combination with ANX2T inhibitor (Fig 4B, compare bar 8 with bars 2 and 7). Taken together, these data indicate that ANX2T inhibitor increases the sensitivity of primary ALL cells to dexamethasone and vincristine when co-cultured with osteoblasts.

To determine the physiological significance of osteoblasts in ALL cell survival *in vivo*, we performed dual immunohistochemistry labeling on femur sections from mice transplanted with NTPL-20 one week prior to sacrifice. Osteoblasts were visualized by staining with osteocalcin, a known osteoblast-specific marker [20] and ALL cells were detected by using CD45 antibody. The CD45 positive ALL cells were seen in close proximity with osteoblasts (Fig 4C), suggesting that the bone marrow osteoblasts are involved in supporting ALL cell survival, similar to their role in HSC maintenance.

### Disruption of interaction between ANX2 and p11 suppresses short-term homing and engraftment of primary ALL cells in immune-compromised mice

**Fig 5:**
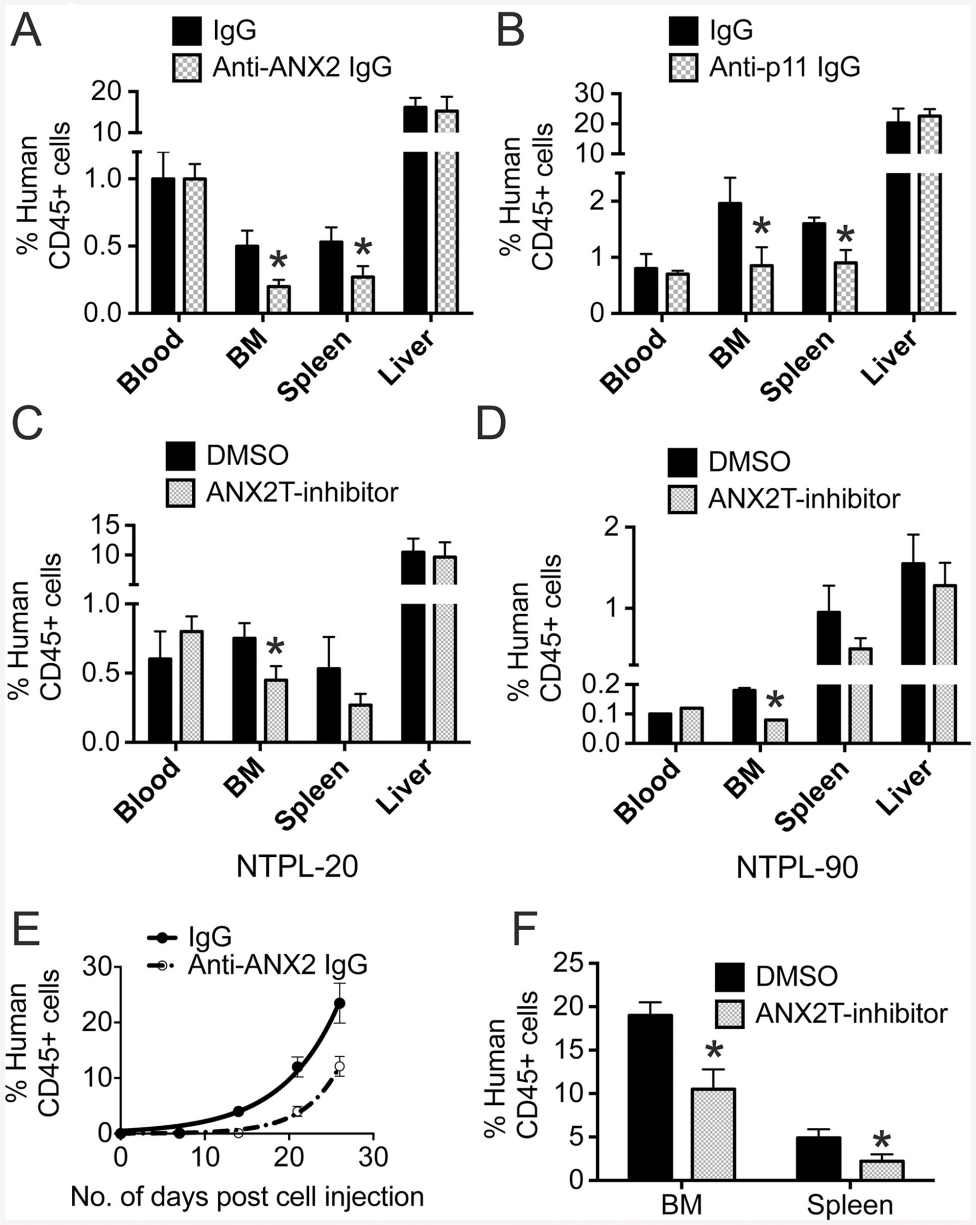
Disruption of interaction between ANX2 and p11 suppresses short-term homing and engraftment of primary ALL cells in immune-compromised mice. **A)** NSG-B2m mice were injected with either anti-ANX2 antibody or an isotype-matched control antibody (IgG) before transplantation of 10 million NTPL-20 cells. Twenty-four hours post cell injection, mice were sacrificed and peripheral blood, spleen, liver, and femurs were harvested. Femurs were flushed to collect bone marrow. The percentage of human cells was detected by flow cytometry. The error bars denote SE of the Mean (n = 6 each). Asterisks indicate statistical significance with P < 0.05. **B)** NSG-B2m mice were injected with either anti-p11 antibody or an isotype-matched control antibody (IgG) before transplantation of 10 million NTPL-20 cells. The percentage of human cells in mouse organs was detected by flow cytometry twenty four hours post transplantation as described in A. The error bars denote SE of the Mean (n = 5 each). Asterisks indicate statistical significance with P < 0.05. **C**) Mice were pre-treated with either ANX2T inhibitor or vehicle before NTPL-20 cell transplantation. The graph shows the percentage of human cells in indicated organs determined twenty-four hours post cell injection. The error bars represent SE of the mean (n = 5). Asterisk denotes P < 0.05. **D**) Mice were pre-treated with either ANX2T inhibitor or vehicle before NTPL-90 cell transplantation. The graph shows the percentage of human cells in indicated organs determined twenty-four hours post cell injection. The error bars represent SE of the mean (n = 5). Asterisk denotes p < 0.05. **E)** The graph shows the increase in the percentage of human cells in mouse blood over time. Error bars denote SE of the mean from five mice each. *P < 0.05. **F)** Percentage of engraftment in bone marrow and spleen in mice at two weeks post transplantation with NTPL-20 cells is shown in the graph. Error bars represent SE of the mean from four mice each. *P < 0.01.

To determine whether ANX2/p11 interaction is involved in short-term homing of primary ALL cells to the bone marrow, NSG-B2m mice were first injected with either anti-ANX2 antibody (ANX2) or an isotype-matched control antibody (IgG) and then NTPL-20 cells were transplanted. Twenty-four hours post cell inoculation, the proportion of human leukemic cells as a percentage of total CD45+ cells was determined in peripheral blood, spleen, liver and bone marrow. The percentage of human leukemic cells in peripheral blood and liver was similar for control and treated mice, indicating that numbers of ALL cells injected were similar for both populations and that the antibody did not have a direct effect on cell viability. The percentage was reduced by 60% in bone marrow isolated from femurs and by 49% in the spleens of treated mice (Fig 5A), indicating that the antibody reduced homing of ALL cells to the bone marrow and spleen. Similar to anti-ANX2 antibody, pre-inoculation with anti-p11 antibody also reduced the percentage of human cells in the bone marrow and spleen by 57% and 44% respectively (Fig 5B). Next, we tested the effect of ANX2T inhibitor on homing of two patient samples (NTPL-20 and NTPL-90) to the bone marrow. Similar to antibody treatment, ANX2T inhibitor also affected homing of ALL cells to the bone marrow (Fig 5C and 5D), confirming the significance of ANX2/p11 interaction in mediating homing of ALL cells.

To determine whether disruption of ANX2/p11 interaction affects tumor engraftment and progression, a cohort of mice treated as described above were then injected with a second dose of anti-ANX2 antibody the day after cell transplantation. The percentage of leukemic cells in blood was determined every week. The percentage of human cells in mouse blood increased rapidly in mice treated with control IgG (doubling time = 4.66 ± 0.29 days), while the mice treated with anti-ANX2 antibody showed a slower progression (doubling time = 2.92 ± 0.03 days) (Fig 5E), suggesting that anti-ANX2 antibody reduced NTPL-20 engraftment and leukemia progression. A separate cohort of mice was treated with two additional doses of vehicle or ANX2T inhibitor following NTPL-20 cell transplantation as described above. Mice were sacrificed two weeks later and bone marrow and spleens were harvested. The percentage of engraftment in bone marrow and spleen was significantly reduced in ANX2T inhibitor treated mice compared to the vehicle treated mice (Fig 5F). Taken together, these data suggest that disengagement of ANX2 and p11 interaction reduces short-term homing of ALL cells and suppresses engraftment of cells in bone marrow and spleen.

## Discussion

**Fig 6:**
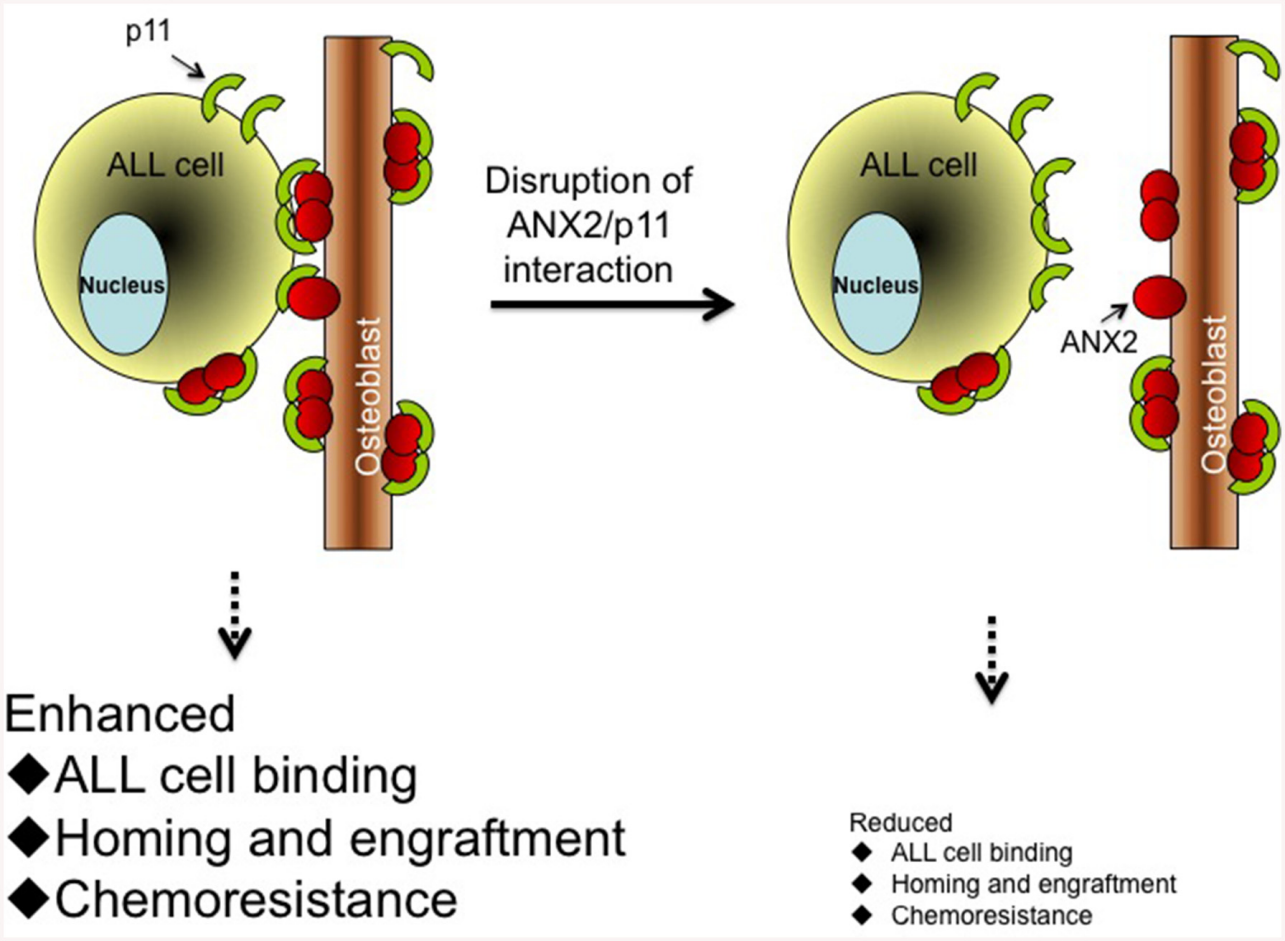
Cartoon summarizing the role of ANX2/p11 interaction in facilitating ALL cell-osteoblast association. The interaction of p11 (on ALL cell surface) to ANX2 (on osteoblast surface) promotes ALL cell binding, homing and engraftment to the bone marrow, and imparts resistance to chemotherapy drugs. Disruption of interaction between these two proteins using ANX2T inhibitor, anti-ANX2 antibody, anti-p11 antibody or p11 knockdown results in reduced binding, homing and engraftment, and sensitizes ALL cells to chemotherapy. Red circles represent ANX2 protein and green semicircles denote p11 protein.

Adhesive interaction between osteoblasts in the bone marrow and ALL cells is believed to facilitate the retention of leukemic cells in the bone marrow niche and provide a sanctuary for the leukemic cells to evade chemotherapy. We discovered that the ANX2/p11 protein-protein interaction between the cell surface of osteoblasts and ALL cells not only mediates adhesion but also regulates homing and engraftment of ALL cells to the bone marrow. Furthermore, disrupting this interaction sensitized ALL cells to chemotherapy, indicating that inhibition of the ANX2/p11 axis has potential therapeutic value to treat ALL, especially when used in combination with existing drugs. A pictorial representation depicting these results is presented (Fig 6).

Cell surface bound ANX2T has been shown to mediate heterotypic cell-cell adhesion between a variety of malignant cell types and host cells within the tumor microenvironment. For example, the role of extracellular ANX2 in mediating the adhesion of liver-metastatic RAW117 large-cell lymphoma cells to hepatic sinusoidal endothelial cells has been demonstrated [21]. Similarly, adhesion of metastatic breast cancer cells to microvascular endothelial cells is mediated by ANX2/p11 interaction [18]. The role of ANX2T is not limited to endothelial cell binding: prostate cancer cells [5] and multiple myeloma cells [6] bind to osteoblasts in the bone marrow through adhesive association between ANX2T and its receptor [22]. ANX2 influences the bone marrow microenvironment via its involvement in osteoclastogenesis [23] and regulates initial adhesion, homing and engraftment of hematopoietic stem cells to the bone marrow endosteal niche following transplantation [4]. However, the role of ANX2/p11 interaction in leukemia was not known. Therefore, the data presented here that ANX2/p11 interaction mediates binding, homing and engraftment of ALL cells to the bone marrow supports the evolving theme that localization of leukemic cells in the bone marrow niche aids their survival and promotes chemoresistance.

Although the disruption of ANX2/p11 interaction reduced ALL cell binding to osteoblasts, and homing and engraftment of ALL cells in the bone marrow, there was no complete abolishment of these effects. It is possible that the levels of the antibody or the inhibitor were not sufficient to completely inhibit the interaction. Another possibility is that similar to the complex and dynamic interplay between osteoblasts and HSCs, other cell adhesion molecules (such as CD44) also contribute to the interactions between osteoblasts and ALL cells [24]. Nonetheless, the significance of ANX2/p11 interaction is highlighted by this study.

How does p11 on the surface of ALL cells interact with ANX2 on osteoblasts? Although it is believed that ANX2 must be complexed with p11 to translocate to the cell surface, p11 alone has been detected on the cell exterior in colorectal cancer cells in the absence of ANX2 [7]. Also, p11 binds to several cell surface proteins other than ANX2 [25], and it is possible that such binding is involved. Alternatively, the low levels of ANX2 present on the surface of ALL cells by flow cytometry could be sufficient to anchor p11 to the cell membrane as described previously [18]. However, the specificity of p11 in mediating adhesive interaction was shown by specific knockdown of p11, which did not alter the levels of ANX2 (data not shown), as reported by other groups [7].

p11 may have additional roles in ALL progression, besides mediating ALL cell binding within the bone marrow niche. It is known to facilitate the activation of plasminogen by tissue plasminogen activators [7]. Thus, p11 on the surface of ALL cells could promote plasmin-mediated degradation of extracellular matrix, thereby facilitating tissue infiltration and homing to the bone marrow. A similar role for p11 in promoting migration and invasiveness has been demonstrated by several studies [26]. Additionally, intracellular p11 also mediates glucocorticoid resistance in MLL-rearranged infant ALL by regulating ANX2 phosphorylation [27]. Studies are in progress in the laboratory to identify other mechanisms by which p11 contributes to leukemogenesis and progression.

Interactions between cell surface molecules that mediate cell adhesion are known to activate signaling pathways such as Akt and ERK1/2 [5, 6]. It is possible that ANX2/p11 interaction similarly induces survival pathways in ALL cells. The observation that ANX2T inhibitor reduced ALL cell survival in the presence of osteoblasts but did not affect cell viability in monoculture indicates that loss of the survival signals in the presence of osteoblasts by ANX2T inhibitor could suppress cell viability. It is also possible that osteoblasts secrete growth inhibitory factors following disruption of ANX2/p11 interaction. Furthermore, ANX2T inhibitor sensitized ALL cells in co-culture to chemotherapeutics, suggesting a synergisism between ANX2T and chemotherapy drugs. Thus, a combination therapy with ANX2T inhibitor and dexamethasone could prove beneficial to prevent relapse and reduce side effects by lowering the dose of chemotherapy. Experiments to test such an approach are currently being conducted in our laboratory. Finally, our data showing elevation of p11 in pediatric B-ALL cells warrants further investigation to determine whether p11 may be used as a diagnostic biomarker for pediatric ALL.
